# Effect of acupuncture and its influence on cerebral activity in perimenopausal insomniacs: study protocol for a randomized controlled trial

**DOI:** 10.1186/s13063-017-2072-7

**Published:** 2017-08-14

**Authors:** Xiao Wu, Wei Zhang, Yuanyuan Qin, Xuguang Liu, Zhengyan Wang

**Affiliations:** 1 Chengdu University of Traditional Chinese Medicine, 37 Shi’er Qiao Road, Jinniu District, Chengdu, Sichuan 610072 People’s Republic of China; 2 Traditional Chinese Medicine Hospital Affiliated to Southwest Medical University, 16 Chunhui Road, Longmatan District, Luzhou, Sichuan 646000 People’s Republic of China; 3 Sichuan Integrative Medicine Hospital, 51 4th section of Renmin South Road, Wuhou District, Chengdu, Sichuan 610041 People’s Republic of China

**Keywords:** Acupuncture, Perimenopausal insomnia, Functional magnetic resonance imaging, Randomized controlled trial, Study protocol

## Abstract

**Background:**

Perimenopausal insomnia is one of the core symptoms of the menopausal transition. Acupuncture is considered to exert a positive effect on restoring the normal sleep–wake cycle. However, there is little intuitive evidence besides evaluation using clinical effectiveness scales. We therefore designed this study, aiming to use more intuitive and reliable detection techniques such as functional magnetic resonance imaging before and after applying acupuncture to provide neuroimaging evidence, as well as to verify the effectiveness with other curative effect indicators.

**Methods/Design:**

This study is a randomized, assessor–statistician-blinded, positive medicine controlled trial involving 40 participants. A total of 40 eligible patients with perimenopausal insomnia will be randomly assigned to two groups in a 1:1 ratio as an intervention group using acupuncture and a control group taking estazolam. Participants in the intervention group will receive six acupuncture treatment sessions per week for 4 consecutive weeks, for a total of 24 sessions during the study. Meanwhile, the medicine control group will be prescribed estazolam 1–2 mg/day to be taken 30 minutes before sleep for 4 weeks. The primary outcome is the Pittsburgh Sleep Quality Index. Secondary outcomes are the micro-movement sensitive mattress-type sleep monitoring system, the Hamilton Depression Scale, and the Hamilton Anxiety Scale. All outcomes will be evaluated before and after treatment. The safety of interventions will be assessed at every visit.

**Discussion:**

The results of this trial, which will be available in 2018, will investigate the impact of acupuncture treating perimenopausal insomnia from assessment of the sleep architecture, hormone level, and emotional-circuit neurological function, and will uncover the effective mechanism of acupuncture regulating the emotional center integrated effect.

**Trial registration:**

Chinese Clinical Trials Register, ChCTR-IPC-16007832. Registered on 26 January 2016.

**Electronic supplementary material:**

The online version of this article (doi:10.1186/s13063-017-2072-7) contains supplementary material, which is available to authorized users.

## Background

Every woman has to undergo the menopausal transition as an inevitable stage of life. During this period, perimenopausal females usually complain of difficulties initiating and/or maintaining sleep with frequent nocturnal and early morning awakenings. Sleep disturbances are often multifactorial in origin and have a significant impact on the health-related life quality. The Study of Women’s Health across the Nation (SWAN) presents that the prevalence of sleep disturbance ascends with increasing age. Recent reviews revealed that the prevalence of insomnia in the perimenopausal women ranges from 39 to 47% [1], while it is approximately 35% in the general population [2].

In addition, with the increasing socioeconomic stress, there are more and more middle-aged women suffering from mood disorders, such as anxiety and depression, which have been noted to be closely related to sleep problems in postmenopausal women [3]. Difficulty in falling sleep has been shown to associate strongly with anxiety, with nonrestorative sleep also correlating strongly with depression, as well as results in anxiety, irritability, and nonrefresh sleep problems which in turn may manifest as depression [4]. Studies by Guidozzi et al. [5] also showed a significant correlation between depression and sleep-related disorders. According to these findings, sleep difficulties and emotional problems interact with and aggravate each other.

Currently, hypnotic medication such as benzodiazepine receptor agonists (estazolam in particular) has been recommended to be the first-choice medicine used routinely for chronic insomnia. Although estazolam is efficacious in the short-term management of insomnia, there is very limited evidence for long-term treatment efficacy [6]. Additionally, various adverse effects related to their use have been reported, including residual daytime sedation, cognitive impairment, and medication dependence [7–10]. Therefore, a large number of patients with insomnia are turning to complementary and alternative medicine such as acupuncture rather than hypnotic medication.

As a chief component of Traditional Chinese Medicine (TCM), acupuncture has been used frequently in the treatment of a wide range of health problems, especially including sleep disturbance and mood disorder, in China and throughout the world [11, 12]. Previous studies certified that acupuncture appears to have an irreplaceable effect in treating insomnia and is able to significantly improve anxiety and depression [13]. Our pilot study also suggested that acupuncture treatment is effective in increasing sleep quality and improving emotional condition [14]. Consequently, it was believed that acupuncture may have great effect on sleep disorders and mood disturbances. Although there are some available studies demonstrating the benefits of acupuncture treating insomnia via evaluation using clinical effectiveness scales, it is still difficult to list more intuitive evidence, such as functional magnetic resonance imaging (fMRI) results.

Considering that acupuncture may have great relevance for modulation of sleep and mood through a potential central neural mechanism, in this trial fMRI—as a more intuitive and reliable detection technique—will be used to investigate the effectiveness and central neural mechanism in acupuncture treatment for perimenopausal insomnia by a comparison with western medicine. Furthermore, we will research the impact of acupuncture treatment for perimenopausal insomnia via changing the sleep architecture and neural function of the emotional neurocircuitry, consequently revealing the integrated effect mechanism of the emotional center function of acupuncture.

## Methods/Design

### Objectives

The study aims to research the influence of acupuncture on perimenopausal insomniacs, such as determining the difference of brain activity and the relevance between the brain and clinical effects, as well as assessing the effect of acupuncture for perimenopausal insomnia.

### Hypothesis

According to the validity of acupuncture on perimenopausal syndrome, insomnia, and emotional disorder, as well as the influence of acupuncture on brain function, we hypothesize that acupuncture will improve the symptoms of perimenopausal insomniacs, and there will be a difference between acupuncture and medicine control based on the fMRI examination.

### Setting

A total of 40 patients diagnosed with perimenopausal insomnia will be divided randomly into two groups (acupuncture intervention group and western medicine control group) through a completely randomized digital table in a ratio of 1:1. This RCT will be conducted in a single center with the assessor and statistician blinded to treatment allocation, and will be carried out in Sichuan Integrative Medicine Hospital, Chengdu, Sichuan, China.

### Patients

#### Recruitment strategies

There will be three prime strategies to recruit participants with perimenopausal insomnia. The first strategy is to recruit participants from the outpatient and inpatient acupuncture and gynecology department of Sichuan Integrative Medicine Hospital. Second, printed recruitment posters will be distributed in public clinics and nearby communities to enroll potential eligible study subjects. Third, we will post advertisements, through the Internet, social software, and so on, to briefly introduce our study, so as to attract possible patients who are willing to participate.

#### Inclusion criteria

Patients meeting all of the following criteria will be enrolled in the study:

1. Patients meeting the diagnostic criteria for primary insomnia in the *Diagnostic and Statistical Manual of Mental Disorders*, fifth edition [15].
2. Patients meeting the diagnostic criteria for perimenopausal syndrome according to the 2011 criteria of the International Menopause Society [16] and the 2001 criteria of the North American Menopausal Society [17].
3. Chief complaint of sleep disorder.
4. Dextromanuality.
5. Female aged between 40 and 55.
6. The Hamilton rating scale for depression (HAMD) score should be 8 points or higher.
7. The Hamilton rating scale for anxiety (HAMA) count should be 6 points or higher.
8. Voluntarily participating in this study with a written informed consent form signed by the participants themselves.

#### Exclusion criteria

Patients meeting any of the following criteria will be excluded from the study:

1. The insomnia is caused by systemic disease (e.g., pain, fever, cough, surgery, etc.) and environmental disturbance.
2. Diagnoses of diabetes mellitus, cardiovascular disease, serious kidney, lung, or liver diseases, hematological disorders, myasthenia graris, or angle-closure glaucoma.
3. With any contraindications of benzodiazepines and acupuncture.
4. Pregnancy or breastfeeding.
5. With metal objects implanted, or any contraindications for fMRI.
6. Patients with any pathological changes in the brain, such as brain tumors, diagnosed on the basis of skull MRI TIW1 and T2W1 routine examination.
7. Patients who have received acupuncture in the past month and are being treated with hypnotic medications.
8. Patients who have taken psychoactive medications or vasoactive medications in the previous 3 months.
9. Alcohol or drug abuse.
10. Any other condition that the investigator judges as likely to make the patient incapable to complete, comply, or unsuitable for the clinical trial.

#### Withdrawal or dropout criteria

1. The participant has an adverse event related to the research.
2. At the participant’s own request.

### Sample size

There is so far still no definite sample size calculation for fMRI research; however, as a result of previous fMRI studies, there should be at least 10 participants in each group to obtain brain imaging with statistical significance [18]. Combining the preceding clinical trial experiences all over the world recently with the withdrawal rate, we plan to enroll a total of 40 participants with 20 patients in each group.

### Ethics

This clinical trial will be carried out in accordance with the Declaration of Helsinki, as well as reviewed and approved by the Medical Ethics Committee Board of Sichuan Integrative Medicine Hospital, Chengdu, Sichuan, China (number: 2016KY-01). All of the participants will be requested to sign the written informed consent form before randomization. The present study is financially supported by the National Natural Science Foundation of China (number: 81503670), and the clinical trial was registered in Chinese Clinical Trials Registry with the identifier number ChCTR-IPC-16007832.

### Procedure

**Fig. 1:**
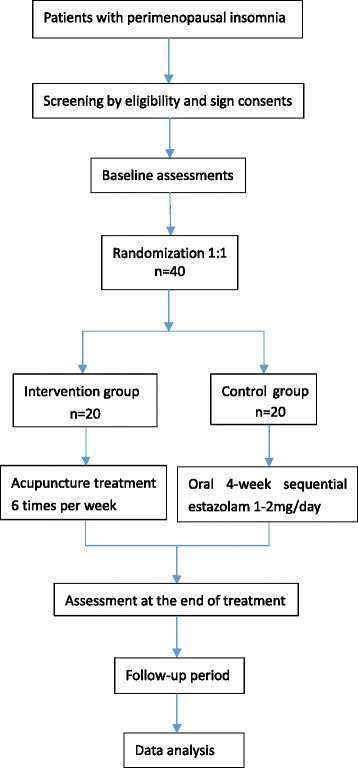
Study flowchart

All of the potential participants will be initially assessed according to the inclusion and exclusion criteria by the physicians. After completing a screening test, the eligible participants will be asked to sign the written informed consent before randomization. The study subjects will then enter a baseline period for 1 week without any treatment. Subsequently, they will receive the intervention in the light of their group allocation for 4 weeks. All of the patients will be required to provide blood specimens for testing relevant indexes, as well as to be evaluated by study-related scales, MSMSMS, and fMRI scan before and after treatment. Two weeks after the end of treatment, patients in both groups will be followed up to assess the curative effect and safety again (Fig. 1).

### Randomization

The eligible participants will be assigned randomly to two groups in a ratio of 1:1. The random number table will be performed by the investigator who has no direct contact with the participants or the assessors to have access to the treatment procedures and outcomes. The randomization number will be sealed in a predetermined randomization opaque envelope. The patients’ screening sequence numbers will be printed on the outside of the envelope, whereas the group names will be printed inside. The envelope containing an allocation sequence number for each patient will be opened right after each patient is verified to meet the eligibility criteria and has signed the written informed consent form. If any error or disclosure regarding randomization occurs, a new randomization sequence will be generated starting from the problematic serial number and applied to subsequent patients.

### Blinding

Considering the significant difference of treatment and the specificity of acupuncture, double blinding of the clinician and patient is unfeasible. However, to guarantee the integrity of this trial, we will strictly conduct randomization allocation and appoint different people to perform therapy, assessment, and statistical analysis according to the principle of blinding.

### Interventions and comparison

#### Rationale for acupuncture (selection of acupoints)

Based on the theory of acupuncture and TCM, perimenopausal insomnia is closely related to the liver. First, the liver plays a vital role in the sleep rhythm and the pathogenesis of sleep problems. Second, emotional disorder, as one of the major factors of insomnia, is also influenced significantly by the liver. According to the basic theory of TCM, the liver governs regulating emotion. Hence, we determined the intervention formula from the liver, and selected the following acupoints as the primary acupuncture points: Baihui (DU20), Sishencong (EX-HN-1), Anmian (EX-HN-54), Ganshu (GB18), Geshu (GB17), Taichong (LI3). Moreover, on the basis of TCM differentiation, subsidiary acupoints are: Taixi (KI3), Quchi (LI11), and Sanyinjiao (SP6) for hyperactivity of liver yang; Xuehai (SP10) and Hegu (LI14) for stagnation of liver qi and blood; Weishu (GB21), Zusanli (ST36), and Liangqiu (ST34) for stagnation of liver qi invading the stomach; Xinshu (GB15), Neiguan (PC6), and Shenmen (HT7) for stagnation of liver qi invading the heart; and Shenshu (GB23) and Taixi (KI3) for liver hyperactivity and kidney deficiency (Table 1).

**Table 1:** Acupuncture points

Syndrome differentiation	Primary point	Additional point
	Baihui (DU20), Sishencong (EX-HN-1)	
	Anmian (EX-HN-54), Ganshu (GB18)	
	Geshu (GB17), Taichong (LI3)	
Hyperactivity of liver yang		Taixi (KI3), Quchi (LI11), Sanyinjiao (SP6)
Stagnation of liver qi and blood		Xuehai (SP10), Hegu (LI14)
Stagnation of liver qi invading the stomach		Weishu (GB21), Zusanli (ST36), Liangqiu (ST34)
Stagnation of liver qi invading the heart		Xinshu (GB15), Neiguan (PC6), Shenmen (HT7)
Liver hyperactivity and kidney deficiency		Shenshu (GB23), Taixi (KI3)

#### Intervention group

The acupuncture points are identified in accordance with the method of point location issued by the World Health Organization (WHO) [19]. After disinfecting the skin on the point local areas, all of the acupoints are punctured by sterile, disposable filiform needles produced by Suzhou Hwato Medical Instruments Co. Ltd (Suzhou, China). The needles are 40 mm in length and 0.25 mm in diameter. The depth of insertion is adjusted based on the standard permissible depth of insertion for each acupoint. Baihui (DU20) will be punctured obliquely with the needle tip towards Qianding (DU21), and Sishencong (EX-HN-1) will be inserted aslant with all the pinpoints towards Baihui (DU20). Needle manipulation will be applied to achieve De Qi sensation, which is manifested as a numb, distended, and aching sensation. The needles will be maintained for 30 minutes in each session, and then removed with clean cotton balls to avoid bleeding. During the treatment period, the needles will be manipulated twice every 10 minutes with intermittent stimulation and each manual performance will last for 10 seconds. Every patient will be treated with acupuncture once per day for 6 consecutive days per week with a 1-day interval and will receive 24 sessions of acupuncture in total during a period of 4 weeks.

#### Control group

The participants in the control group will be prescribed oral administration of estazolam tablets (1 mg 30 minutes before sleep once per day for 4 weeks; 2 mg for severe insomnia) which are commonly used for insomnia. The estazolam tablets are produced by Forth Medicine Pharmacy CO.., Ltd (Changzhou, China) (Approval No.: H32020450).

#### fMRI examination procedure

The MRI examination (Signa 3.0 T Magnetic Resonance Imaging Scan; GE General Electric Company) will be performed to collect brain images offering more visualized proof. The MRI protocol for this study consists of the following parameters: high-resolution three-dimensional T1 weighting brain volume (Bravo) sequence scanning: repetition time (TR) of 8.16 ms, echo time (TE) of 3.18 ms, flip angle 7°, 256 × 256 mm^2^ field of view (FOV), voxel size of 1 × 1 × 1 mm^3^, 180 sections of 1.0-mm thickness. Before scanning, the subjects will be requested to keep quiet and their eyes closed, especially not to think something and to avoid sleep. In order to ensure the participants stay awake, the researcher will inquire of the subject whether they are awake during and after the scanning procedure. Both of the intervention groups will be examined twice (before and after treatment).

#### fMRI data processing

Functional MRI data will be processed by statistical parametric mapping (SPM8) of MATLAB2009b, DPARSF, and Freesurfer (http://surfer.nmr.mgh.harvard.edu/) software packages. Original data will be corrected; physiological artifacts, slice timing, affine head motion, and nonbrain extraction will also be implemented. Moreover, spatial smoothing and temporal filtering will be applied. Cortical surface reconstruction will be performed to improve structural–functional coregistration using FreeSurfer’s register tool (http://surfer.nmr.mgh.harvard.edu/), and then the cortex thickness will be calculated. The cortex thickness, superficial area, and volumetric of every group will be compared using a paired *t* test (*p* < 0.001, cluster size > 100 vertices). The cortex thickness, the local consistency changes of the brain region, and clinical outcomes with significant statistical differences will be analyzed by multiple regression analysis. Then we will acquire the brain regions correlated with an acupuncture clinical effect via analyzing the correlation index values *R* and *P* with the change of cortex thickness, ReHo of brain region, and clinical outcomes.

#### Outcome assessments/measurement

All of the outcomes will be assessed twice before and after the intervention treatment.

### Primary outcome

#### Pittsburgh Sleep Quality Index

The Pittsburgh Sleep Quality Index (PSQI), a self-rated questionnaire that evaluates sleep quality and disturbance over 1 month [20], is applied as the primary outcome measurement for clinical effectiveness assessment. The constructive validity and reliability of the PSQI to measure changes of sleep quality have been confirmed in previous studies all over the world. It is widely used to measure response to treatment in research studies and clinical trials. The PSQI is comprised of 19 items which consist of the following seven component scores: subjective sleep quality, sleep latency, sleep duration, sleep efficiency, sleep disturbance, use of hypnotic medication, and daytime dysfunction. Each item is weighted equally on a 4-point scale, from 0 to 3, with 0 indicating no dysfunction and 3 indicating the worst dysfunction. The seven component scores are then added to provide a global PSQI score. In general, a lower global score reflects a better sleep quality [21].

### Secondary outcome

#### Micro-movement sensitive mattress-type sleep monitoring system

All of the study subjects will be measured for two nights (baseline: after randomization and before treatment, after the completion of treatment) in a sleep laboratory with a sound-attenuated, light-controlled, and temperature-controlled room. The TDM-6000 micro-movement sensitive mattress-type sleep monitoring system (MSMSMS) will be conducted to precisely obtain the continuous dynamic physiological parameters of the patients at night, as well as to compare the proportion of deep sleep, light sleep, and rapid eye movement (REM) sleep during the sleep period. During assessment, patients will be permitted to sleep optionally based on their habitual sleep time, with the recording time ranging from the moment of patient lying on the bed to the time of getting up. Sequentially, we can analyze the sleep architecture/structure, and accurately identify the various kinds of sleep disorders.

#### Hamilton Depression Scale and Hamilton Anxiety Scale

Changes in levels of mood disorder will be measured on the Hamilton Depression Scale (HAMD) and the Hamilton Anxiety Scale (HAMA).

There are three types of HAMD scale, each with 17, 21, or 24 items. The 24-item HAMD scale will be used to evaluate the depressive condition in this trial. According to the score standard, a normal score is <8; a score ≥8 and <20 indicates that the patient has mild depression; a score ≥20 and ≤35 indicates that the patient has moderate depression; and a score >35 indicates that the patient has severe depression. Typically, the higher score reflects the more severe depression [22].

To assess anxiety, there is a 14-item self-rating questionnaire for evaluating the anxiety condition of last week. Based on the score standard, answers are scored on a 0–4 scale, with 0 indicating no symptoms and 4 indicating the worst anxiety condition, with a range in total score from 0 to 56.

In general, the normal threshold is 6 points; scores ranging from 0 to 5, from 6 to 13, from 14 to 20, from 21 to 28, and as 29+ are typically used to rate anxiety as none, mild, moderate, severe, and extremely severe, respectively. In general, the higher the score, the more severe the anxiety is.

In this study, both HAMD and HAMA will be evaluated twice (before and after treatment).

### Follow-up

The follow-up assessment, which is designed to evaluate the clinical curative effect and long-term effects of acupuncture, as well as safety of the study, will be carried out 2 weeks after the end of treatment. Patients will document the details of clinical symptoms and adverse events, and send the relevant information to researchers via call, email, or short message service.

### Statistical analysis

In this study, statistical analysis will be performed by a professional statistician blinded to the whole trial process using SPSS19.0 statistical software, and based on the intention-to-treat principal and preprotocol population. All demographic, baseline characteristics, curative effects, and fMRI images of the subjects will be analyzed with different approaches.

In terms of the data description, the enumeration data/quantitative data will be expressed with/as a percentage or proportion, while the measurement data/qualitative data will be represented as average and standard deviation. On this occasion of normal distribution and homoscedasticity, one-way analysis of variance (ANOVA) will be applied to identify differences in measurement data/qualitative data of comparison. Other covariates will be considered for further revision and if necessary stated in the statistical analysis project prior to data lock. Similar analysis of covariance (ANCOVA) models will used for secondary analysis to adjust baseline characteristics. A *q* test (Newman–Keuls) will be used for pairwise comparison. In case of nonnormally distributed data and heteroscedasticity data, a nonparametric test (Kruskal–Wallis *H* test) will be performed, and a rank sum test (Nemenyi) will be conducted to identify difference between groups. The enumeration data/quantitative data will be compared by chi-square test, while classification data will be evaluated with a rank sum test. In addition, the exploratory analysis will be implemented to describe group means over time. A two-sided test will be applied for available data and *p* < 0.05 will be taken as significant for each outcome. Missing data will be explored and if necessary multiple imputations will be utilized.

### Safety monitoring

To exclude patients who have severe heart, liver, and kidney diseases, all participants will receive routine tests of blood, urine and stool, liver function, kidney function, and electrocardiogram before randomization. Meanwhile, aiming to evaluate the side effects of both interventions, participants will receive these examination items again after the end of treatment.

Possible adverse events due to acupuncture include needle fainting, needle sticking, local infections, subcutaneous hematoma, and so forth. Any adverse events or reactions occur during the study process will be addressed properly and analyzed by the investigator. Serious adverse events associated with the trial should be reported to the principal investigator immediately. All unexpected and unintended responses will be documented as adverse events by the researcher at every visit, even if they are not necessarily related to the acupuncture intervention. The details of adverse events will be carefully recorded in the CRF by the corresponding research staff.

### Quality control

To ensure the control group’s compliance, participants will receive the estazolam tablets of 1 week dose at the face-to-face interview every week, and sign on the medicine record of the CRF. In addition, our researcher will communicate with them every day to visit their medication via call, short message, or Internet connect tools.

**Fig. 2:**
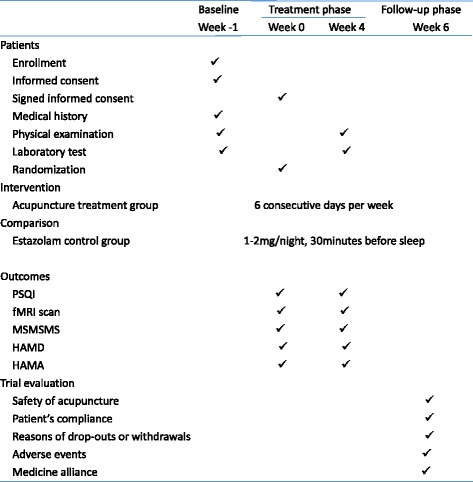
Trial processes chart. *PSQI* Pittsburgh Sleep Quality Index, *fMRI* functional magnetic resonance imaging, *MSMSMS* micro-movement sensitive mattress-type sleep monitoring system, *HAMD* Hamilton Depression Scale, *HAMA* Hamilton Anxiety Scale

All of the acupuncturists who take part in this trial have gained medical licenses in China and have been well trained according to the trial’s procedure under the guidance of senior acupuncturists. All of the investigators were qualified to perform this study after they participated in special training classes about this trial and were tested to well understand the research. For instance, they were trained to use the randomization number table, to fill in the case report form, to identify the correct acupoints, to manipulate the needles, and so on. Additionally, to ensure the quality of the study, the clinical monitors will check the process of the trial and document the details of the processes once a week. Moreover, monitors nominated by the principal investigator will check the accuracy and validity of the original data from the clinical center. Regular meetings will be held to consider the recruitment rate, adverse events, protocol violation, difficulties and problems emerging during the study, and so forth (Fig. 2).

## Discussion

This proposed study is a randomized, assessor–statistician-blinded, controlled clinical trial which is sponsored and financially supported by the National Nature Science Foundation of China. The purposes of this program are to evaluate the efficacy and safety of acupuncture for treating perimenopausal insomnia by assessing the sleep architecture, hormone level, and emotional-circuit neurological function, and to identify the effective mechanism of acupuncture regulating emotional center integration.

In the view that poor sleep quality during menopausal transition is closely related with mood disorder, the formula of acupuncture adopted in this trial will be based on syndrome differentiation from the liver. In recent years, there have been some published reports suggesting that treating insomnia according to syndrome differentiation from the liver is significantly effective [23, 24]. Some researchers investigated 3380 patients with insomnia in Shanghai and demonstrated that there are six basic types of insomnia: with hyperactivity of liver yang, stagnation of liver qi and blood, stagnation of liver qi invading the stomach, stagnation of liver qi invading the heart, fire derived from stagnation of liver qi, and wind derived from stagnation of liver qi [25]. On the basis of the syndrome differentiation from the liver, the previous study treated 2262 patients with sleep disturbance according to the therapeutic principle of soothing the liver, relieving depression, activating blood, and calming the mind; consequently it achieved a good curative effect [26]. Our pilot study also demonstrated that using acupuncture to treat perimenopausal insomnia from the liver is reliable and effective [14]. We therefore determined to design this study with the acupuncture intervention formula based on liver differentiation to provide evidence for the effects from clinical curative effect outcomes and the potential central mechanism.

Besides using the PSQI, MSMSMS, HAMD, and HAMA to assess the clinical curative effect, we choose fMRI technology to reveal the brain effective mechanism of acupuncture treatment for perimenopausal insomnia, as well as to provide clear, valid, and visual evidence.

There are several available evidence sources about acupuncture treating insomnia, perimenopausal syndrome, and emotional disorders [27–38]. Previous studies revealed the hippocampus volume of insomniac decreased notably [39–43] and acupuncture can improve insomnia significantly and change the brain region activity. Consequently, we hypothesize that acupuncture can improve perimenopausal insomniac with emotional problems via change in the correlated brain region’s activity, and we designed this study to assess the effects and research the influence of acupuncture for perimenopausal insomniacs.

There are also some accessed findings about several brain regions which are concerned with emotional problems. Previous studies suggested that the cerebral limbic system and the surrounding structure are the brain regions which are strongly linked with the affective state, as well as adjusted by frontal lobe, temporal cortex, and subcortex. Recent neuroimaging studies have implicated that several brain regions, including the orbitofrontal cortex (OFC), ventral medial prefrontal cortex (vmPFG), amygdale, hypothalamus, brainstem, cingulated cortex, thalamus, hippocampus, sensory cortex, and so on, play a key role in emotional regulation [44–46]. Meanwhile, other studies investigating the plasticity of emotional neurocircuitry and mood disorders also provide substantial support for the viewpoint that some brain regions, such as the prefrontal cortex, amygdale, hippocampus, and thalamus, are the critical sections of emotional neurocircuitry and exert an important effect on emotional regulation [47, 48]. Sleep disturbances may contribute to the weakened emotional regulation of the prefrontal cortex. Long-term insomniacs tend to build an inner emotion mechanism to relieve the stress of insomnia [49]. Moreover, findings of a cerebral functional imaging study declared that when the subcortex and autonomic nerve system are inhibited by the prefrontal cortex, a bad affective state including anxiety and depression will be generated [50, 51]. Some research considered that the amygdale may impact the plasticity of the hippocampus and thalamus [52, 53], whereas its plasticity is also influenced by regulation of the prefrontal cortex, which will inhibit negative emotion by connecting with the amygdale [54]. Given that the amygdale may be closely related with sleep disorder, previous studies revealed that when a primary insomnia patient turns to a sleep state from waking status there are abnormal activity brain regions including the amygdale, hippocampus, thalamus, and prefrontal cortex [55, 56]. Based on the possibility that the acupuncture signal may stimulate the central integration neural signal and improve emotional disorder, and the findings that there may be abnormal nerve function in the emotional neurocircuitry of insomniacs, it is hypothesized that the emotional neurocircuitry and emotional center cortex may integrate and respond to the acupuncture signal; during this procedure, central regulation of the prefrontal cortex and functional connection of the amygdale may play an important role. Thus, after the sleep-related center responded/answered acupuncture signal, positive regulation of the sleep architecture and mood disorder is able to improve sleep difficulties.

Nevertheless, there are also limitations in this study. Concerning the unfeasible blinding to participants, one limitation is the possibility of a high risk of bias regarding blinding by unconcealed allocation. We therefore strictly conduct randomization and separate the acupuncturist, assessor, and statistician. Meanwhile, patients divided into the acupuncture intervention group may have more opportunities to make contact with their acupuncturists and establish a good doctor–patient relationship rather than those of the western medicine control group, which is likely to improve the therapeutic effect [57]. Hence, we will maintain close contact with patients in the control group by telephone, Internet, and so on, to minimize this potential bias.

In conclusion, the results of this trial are expected to confirm whether an acupuncture formula based on liver differentiation is effective in relieving perimenopausal insomnia. Combining neuroimaging with a neurophysiology method, the relevant outcomes are predicted to verify the central mechanism of acupuncture treatment. We believe that the results will exert a positive effect on acupuncture treatment for perimenopausal insomnia (Additional files 1 and 2).

## Trial status

This trial is currently in the recruitment phase.

## Additional files

Additional file 1: SPIRIT 2013 checklist. (DOC 124 kb)

Additional file 2: Consent form. (DOC 114 kb)

## Abbreviations

TCM: Traditional Chinese Medicine
ANOVA: Analysis of variance
PSQI: Pittsburgh Sleep Quality Index
MSMSMS: Micro-movement sensitive mattress-type sleep monitoring system
HAMD: Hamilton Depression Scale
HAMA: Hamilton Anxiety Scale
fMRI: Functional magnetic resonance imaging
WHO: World Health Organization
TR: Repetition time
TE: Echo time
